# Three-dimensional reconstruction of rat sperm using volume electron microscopy: Morphological process of spermiogenesis revealed by ATUM-SEM

**DOI:** 10.3724/abbs.2024144

**Published:** 2024-09-03

**Authors:** Jiazheng Liu, Limei Lin, Lina Zhang, Hongtu Ma, Xi Chen, Keliang Pang, Linlin Li, Hua Han

**Affiliations:** 1 School of Future Technology University of Chinese Academy of Sciences Beijing 101408 China; 2 State Key Laboratory of Multimodal Artificial Intelligence Systems Institute of Automation Chinese Academy of Sciences Beijing 100190 China; 3 Transdisciplinary Platform of Functional Connectome and Brain-inspired Intelligence Chinese Academy of Sciences Beijing 101499 China; 4 Aging and Longevity Institute & Institute of Biological Science Zhongshan Hospital Fudan University Shanghai 200032 China

**Keywords:** spermiogenesis, spermatid, three-dimensional reconstruction, ATUM-SEM

## Abstract

Three-dimensional (3D) reconstruction serves as a crucial instrument for the analysis of biological structures. In particular, a comprehensive and accurate 3D ultrastructural examination of rat sperm is vital for understanding and diagnosing male fertility issues and the underlying causes of infertility. In this study, we utilize the automated tape-collecting ultramicrotome scanning electron microscopy (ATUM-SEM) imaging technique, which is a highly effective method for 3D cellular ultrastructural analysis. Our findings reveal that during spermiogenesis, the volume of the nucleus significantly decreases, shrinking to just 10% of its original size. The acrosomal vesicles derived from the Golgi apparatus converge and elongate along the spermatid nucleus. These vesicles then attach to the nucleus via a cap-like structure, thereby defining the head side of the spermatozoa. In the initial stages of spermiogenesis, the mitochondria in spermatids are distributed beneath the cell membrane. As the process progresses, these mitochondria gradually migrate to the sperm tail, where they form the mitochondrial sheath. This sheath plays a crucial role in providing the energy required for the movement of the sperm. In addition, we reconstruct the mRNA-stroring structure-chromatoid body in sperm cells, which are cloud-like or net-like structures in the cytoplasm. The precise and comprehensive nature of 3D ultrastructural examination allows for a deeper understanding of the morphological process of spermiogenesis, thereby contributing to our knowledge of male fertility and the causes of infertility. Our research has significantly advanced the understanding of the 3D ultrastructure of sperm more comprehensively than ever before.

## Introduction

Spermiogenesis is the final stage of spermatogenesis, during which haploid, round spermatids undergo a series of morphological and molecular transformations to become mature spermatozoa. This process involves significant changes in cell morphology, including the formation of the sperm head, neck, and tail structures, and is accompanied by profound alterations in the chromatin structure within the nucleus [ 1– 3]. The acrosome, a specialized organelle that covers the anterior part of the sperm head, is formed from Golgi-derived vesicles that coalesce and elongate along the anterior end of the nucleus. The mature acrosome is a cap-like structure that covers approximately two-thirds of the nucleus surface and is essential for the ability of sperm to penetrate the egg’s outer layers during fertilization [ 4, 5]. The mitochondria in spermatids initially localize beneath the cell membrane, and as spermiogenesis progresses, they migrate toward the developing sperm tail. These mitochondria undergo changes in volume and structure, becoming smaller, and eventually wrap around the central axoneme to form a helical mitochondrial sheath. This sheath is critical for providing the energy required for sperm motility [ 4, 6, 7]. Additionally, the centrioles in spermatids migrate and contribute to the formation of the central axoneme of the sperm tail, which is the core structural component of the axoneme. After the complex morphological changes that occur during spermiogenesis, the newly formed spermatozoa are released into the lumen of the seminiferous tubules. They are then transported to the epididymis, where they undergo further maturation processes that are essential for acquiring motility and fertilization capability [ 8, 9].

Our current understanding of spermiogenesis has been largely informed by traditional light microscopy and conventional electron microscopy (EM) techniques [ 4, 9]. These methods, however, have inherent limitations, such as the lack of true three-dimensional (3D) ultrastructural detail, which fails to accurately reflect the complete process of spermiogenesis. Recent advancements in EM-based techniques for 3D ultrastructural reconstruction, particularly in the nervous system, have led to the development of a new category of EM known as volume EM. Volume EM encompasses serial-section electron microscopy (ssET) [10], serial block face scanning electron microscopy (SBEM) [11], focused ion beam scanning electron microscopy (FIB-SEM) [12], automated tape-collecting ultramicrotome scanning electron microscopy (ATUM-SEM) and automated serial-section collection with high-throughput transmission EM, such as GridTape [ 13, 14].

To reveal the real architecture of sperm, we recently applied three-dimensional reconstruction images of sperm, which were based on serial cross-sectional images acquired via the ATUM-SEM technique [15]. This technique enables the acquisition of intricate ultrastructural details during spermiogenesis, including the formation of the acrosome and the generation of mitochondrial sheets, among other processes. This approach allowed us to delineate the morphological transformation of sperm with unprecedented clarity and precision.

## Materials and Methods

### Animal experiment

Three 8-week-old male wild-type SD (Sprague–Dawley) rats were bred in the animal facility of Tsinghua University. All the rat experiments were carried out according to the recommendations of AAALAC (Association for Assessment and Accreditation of Laboratory Animal Care International). The IACUC (Institutional Animal Care and Use Committee) of Tsinghua University approved the animal experiment protocol (15-LB5) used in this study. The rats were maintained on a standard 12 h light/12 h dark cycle. Food and water were provided ad libitum unless otherwise noted.

### Sample preparation

To guarantee a higher contrast and better electrical conductivity of the samples, we adopted the OTO approach for sample preparation, which was combined with other classical heavy metal staining techniques [ 16, 17].

The rats were anesthetized via isoflurane inhalation and then subjected to transcardial perfusion for fixation. Initially, the blood was rapidly flushed with 0.1 M phosphate buffer (PB). This was followed by quick perfusion with a solution of 2% paraformaldehyde (PFA) and 1.25% glutaraldehyde (GA) in 0.1 M PB. The perfusion with the fixative was then slowed down and continued for no longer than 20 min. After perfusion, the testes were removed. Small sample blocks, approximately 1 mm ^3^ in size, were trimmed from the testes and postfixed in a solution of 4% PFA and 2.5% GA in 0.1 M PB. Postfixation was carried out with 2% osmic acid prepared in 0.1 M PB for 90 min. This was immediately followed by treatment with 3% potassium ferrocyanide without any intermediate cleaning. After another 90 min, the samples were thoroughly washed with deionized water (ddH _2_O). The samples were subsequently treated with 1% Thiocarbohydrazide or 45 min in an oven set at 30°C. Post-Thiocarbohydrazide treatment, the samples were extensively washed with ddH _2_O and then treated with a fresh solution of 2% aqueous osmic acid for 90 min. The samples were then transferred to a new centrifuge tube for washing. This was followed by an overnight treatment with 2% uranyl acetate at 4°C. Importantly, all steps involving heavy metal staining should be conducted under light-protected conditions. After soaking in uranyl acetate, the samples were transferred to a 50°C oven for 30 min. The process then began with ethanol gradient dehydration, which was conducted on a horizontal shaker. The sample subsequently underwent Epon 812 epoxy infiltration on a horizontal shaker. The sample was immersed in epoxy/acetone solutions of increasing epoxy concentration: 1:1, 2:1, and 3:1, each for a duration of 4–8 h. Pure epoxy was then infiltrated twice under negative pressure, and each infiltration lasted 4–8 h. The final step was polymerization. The samples were placed in embedding plates and processed at 65°C for 24 h.

### Collecting serial sections via tape

The sections were collected via an RMC Powertome ultramicrotome, complemented by an ATUMtome automated collection machine (RMC PowerTome, Phoenix, USA) [18]. Initially, the sample surface underwent a refinement process. Next, the ATUMtome device was employed to automatically collect the sections. This device is designed to gather sections at the liquid level of ultramicrotome diamond knives, which are then transferred onto Kapton sample-bearing rolls via a conveyor mechanism. For this particular study, the section thickness of the testicular tissue was set to 60 nm.

In the process of bookbinding slices, rolls of the slices were cut to the required length and then adhered to a silicon wafer with a diameter of 10 cm via double-sided conductive tape. The assembly was then heated in an oven at 60°C for more than 30 min to facilitate electrical conductivity treatment. Next, the silicon chips containing the slices were placed in a high vacuum coater (DE, Leica EM ACE600) for carbon spraying. The thickness of the carbon layer applied was 6 nm. This carbon coating enhances the electrical conductivity of the sample surface and minimizes the accumulation of charge.

### SEM image acquisition

The imaging process was conducted via a custom-built, high-throughput scanning electron microscope (SEM)-focused beam tomography (FBT) system. This system was integrated with proprietary software for batch positioning of regions of interest (ROI). This software, developed in-house, enables the precise location of ROIs across all slices on a wafer and sequences these ROIs on each slice, facilitating efficient imaging. The imaging process involved a total of 639 slices, each with an imaging volume of 870×760 μm ^2^. The SEM was set to an accelerating voltage of 4 kV. The dwell time for imaging was 43.6 ns, and the resolution achieved was 5 nm. The total volume of imaging data generated was 22.03 TB. This setup and process allowed for efficient and high-resolution imaging of the ROIs, providing valuable data for further analysis.

### Organelle segmentation and 3D reconstruction

We performed precise alignment of extensive datasets via proprietary software developed on our platform. This ensured the accurate overlay of bioinformatic coordinates between consecutive tissue sections. Our aim was to reconstruct the three-dimensional architecture of cells and organelles as accurately as possible.

Organelle segmentation: Precise segmentation of organelle structures is essential for three-dimensional (3D) reconstruction. In our review, we focused on 3D reconstructions of three distinct cellular states observed during spermiogenesis, categorized as the Golgi phase, acrosome cap phase, and acrosomal phase. Owing to the limited data available, manual annotation was conducted via Fiji-TrakEM software.

3D reconstruction: Data annotated via Fiji-TrakEM2 were imported into the Amira software suite for 3D reconstruction and rendering.

## Results

**Figure 1:**
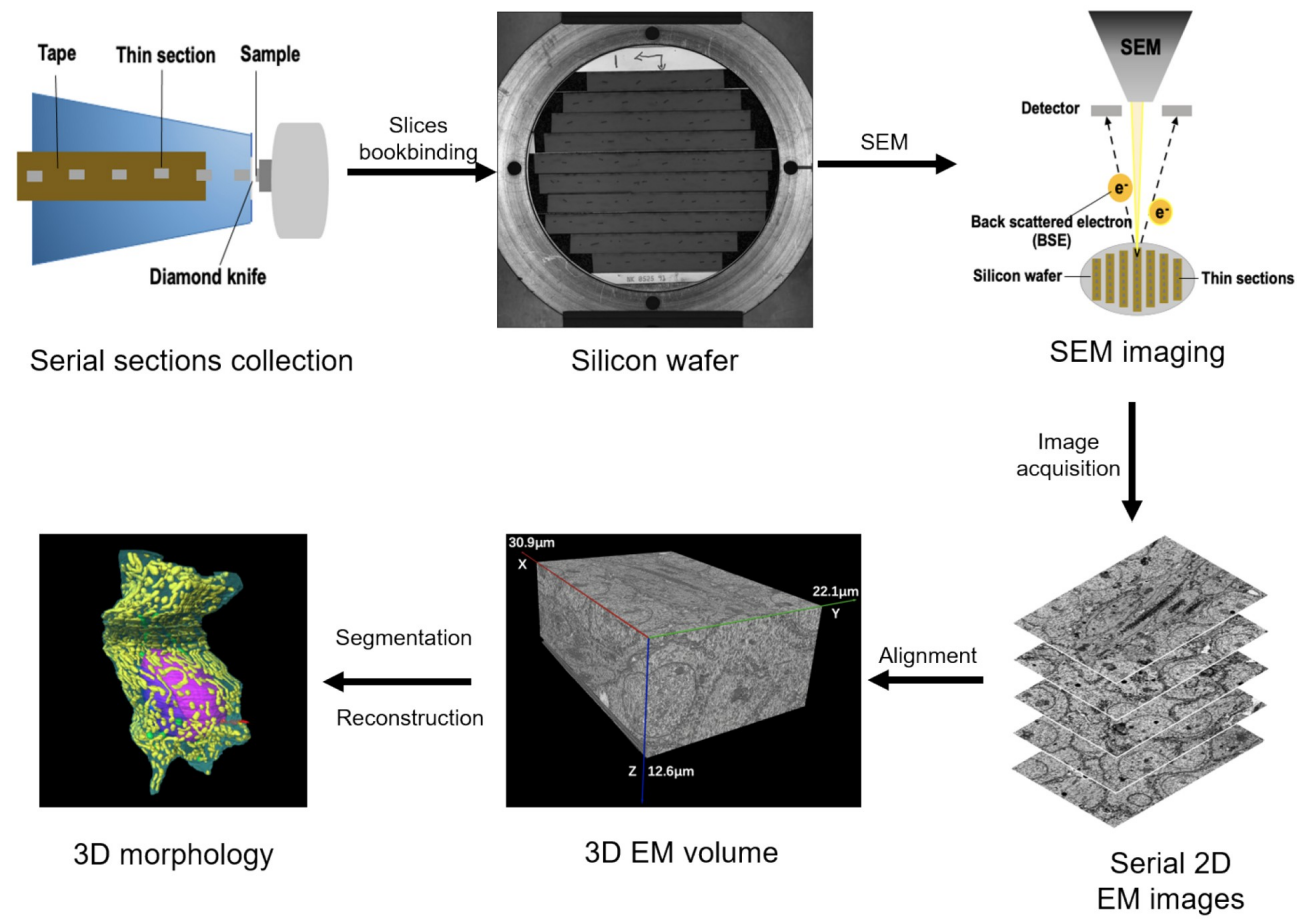
Figure 1 Pipeline of 3D reconstruction for mouse spermatids at different phases First, serial sections of mouse testis EM samples were collected on tape by ATUM. Then, the tape was segmented and attached to the silicon wafers, followed by carbon coating. Next, the serial sections were imaged via SEM, and a coarse-to-fine alignment method was applied to obtain a fine 3D image stack. Finally, the spermatids at different stages were segmented and 3D visualized via Amira software.

We utilized an automatic tape-collecting ultramicrotome (ATUM) to divide a rat testis electron microscopy (EM) sample into 3000 slices, each with a thickness of 60 nm, as shown in Figure 1. The tape was subsequently trimmed into strips and mounted onto silicon wafers in preparation for carbon coating. The sections were then imaged via a scanning electron microscope (SEM) equipped with backscattered electron detection (BSE) at an incident electron energy of 4 keV. The acquired images boasted a resolution of 5 nanometers in both the X- and Y-axes, enabling clear differentiation of the membrane boundaries. Following image alignment, we successfully reconstructed three volumes of the rat testis. Using Amira software, we performed manual segmentation and reconstruction of cellular structures, including mitochondria, the Golgi apparatus, nuclei, and cell membranes ( Figure 1).

### Dimentional morphology of Golgi-phase spermatids

**Figure 2:**
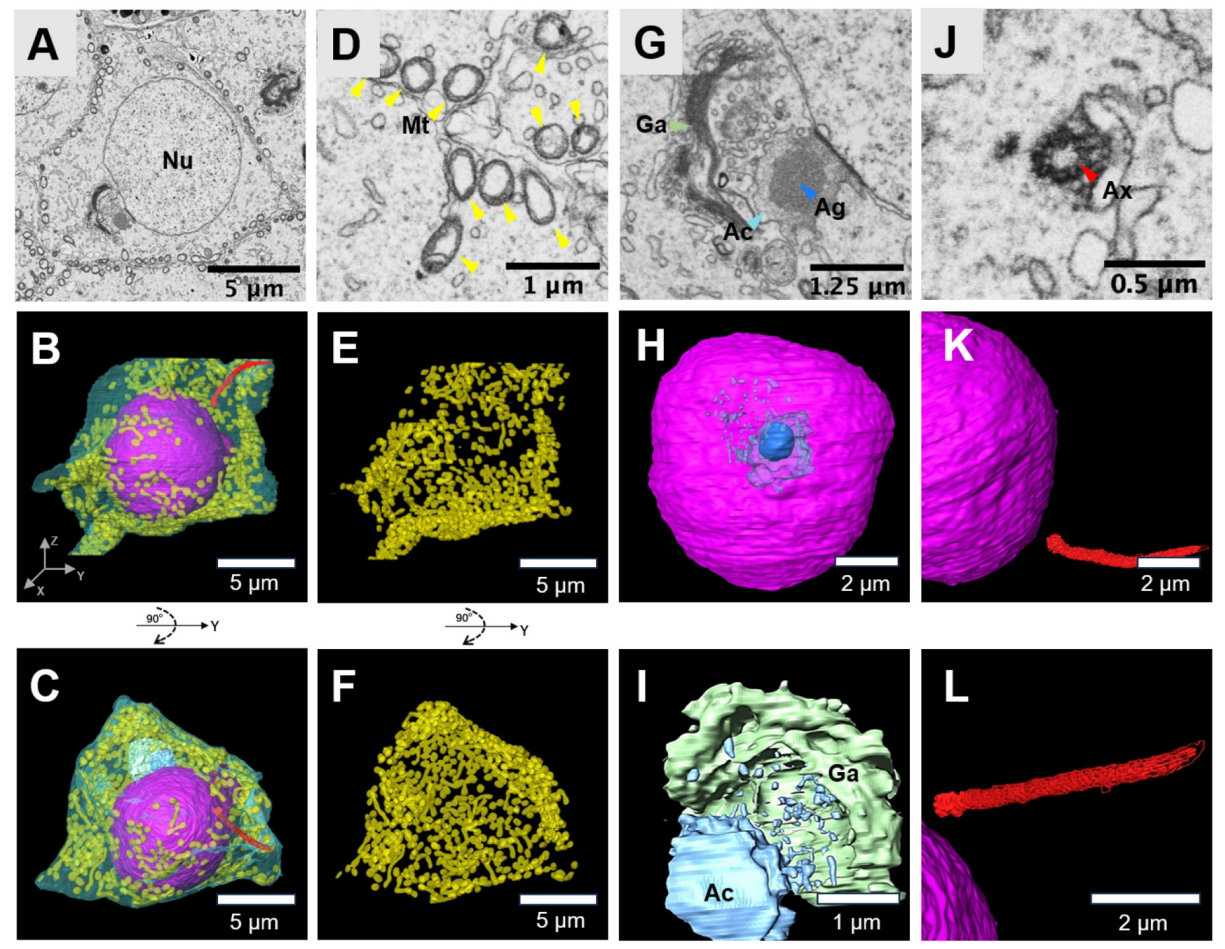
Figure 2 Three-dimensional morphology of Golgi-phase spermatids (A) Typical SEM image of a Golgi-phase spermatid. Scale bar: 5 μm. (B,C) 3D reconstruction of the cell membrane (transparent emerald green), mitochondria (yellow), acrosome cap (light blue), acrosomal granule (dark blue), Golgi apparatus (green), axoneme (red) and nucleus (purple) of the spermatid. Scale bar: 5 μm. (D) SEM image of the mitochondria in the spermatid. Scale bar: 1 μm. (E,F) 3D reconstruction of the mitochondria. Scale bar: 5 μm. (G) SEM image of the Golgi apparatus. Scale bar: 1.25 μm. (H) 3D reconstruction of the acrosome cap, acrosomal granules and nucleus. Scale bar: 1.25 μm. (I) 3D reconstruction of the Golgi apparatus and acrosome cap. Scale bar: 1 μm. (J) SEM image of the axoneme. Scale bar: 0.5 μm. (K,L) 3D reconstruction of the axoneme. Scale bar: 2 μm. Mitochondria, Mt; acrosome cap, Ac; acrosomal granule, Ag; Golgi apparatus, Ga; axoneme, Ax; nucleus, Nu.

The Golgi phase is the beginning of spermiogenesis. Using volume electron microscopy, we reconstructed Golgi-phase spermatids and their organelles, including mitochondria, the nucleus, the Golgi apparatus, and acrosomal vesicles ( Figure 2A–C). In this phase, vesicles coated with proteins bud from the trans-Golgi network, aggregate, and fuse to form the proacrosomal vesicle, which then attaches to the nucleus ( Figure 2A). Concurrently, the morphology and distribution of mitochondria undergo significant changes. The mitochondria, which are initially round in shape, transform into swollen and condensed forms ( Figure 2D). The mitochondria, which are primarily ovoid in shape, were predominantly located beneath the cell membrane ( Figure 2E,F). Pre-acrosomal vesicles, which originate from the Golgi complex, aggregated on the nucleus surface and fused to form large acrosomal vesicles. The area of the nucleus surface in contact with the acrosomal vesicle subsequently forms the head of the spermatozoa. Concurrently, dense proacrosomal granules, which are also derived from the Golgi, fuse to form a single acrosomal granule located in close proximity to the nucleus ( Figure 2G–I). Additionally, one of the centrioles from the centrosome migrates to the posterior pole of the spermatid, a process that mirrors the formation of the axoneme ( Figure 2J–L). The mitochondria then migrate toward the middle section of the sperm tail, initiating the formation of the mitochondrial sheath.

### Dimentional morphology of spermatids at the acrosome cap stage

**Figure 3:**
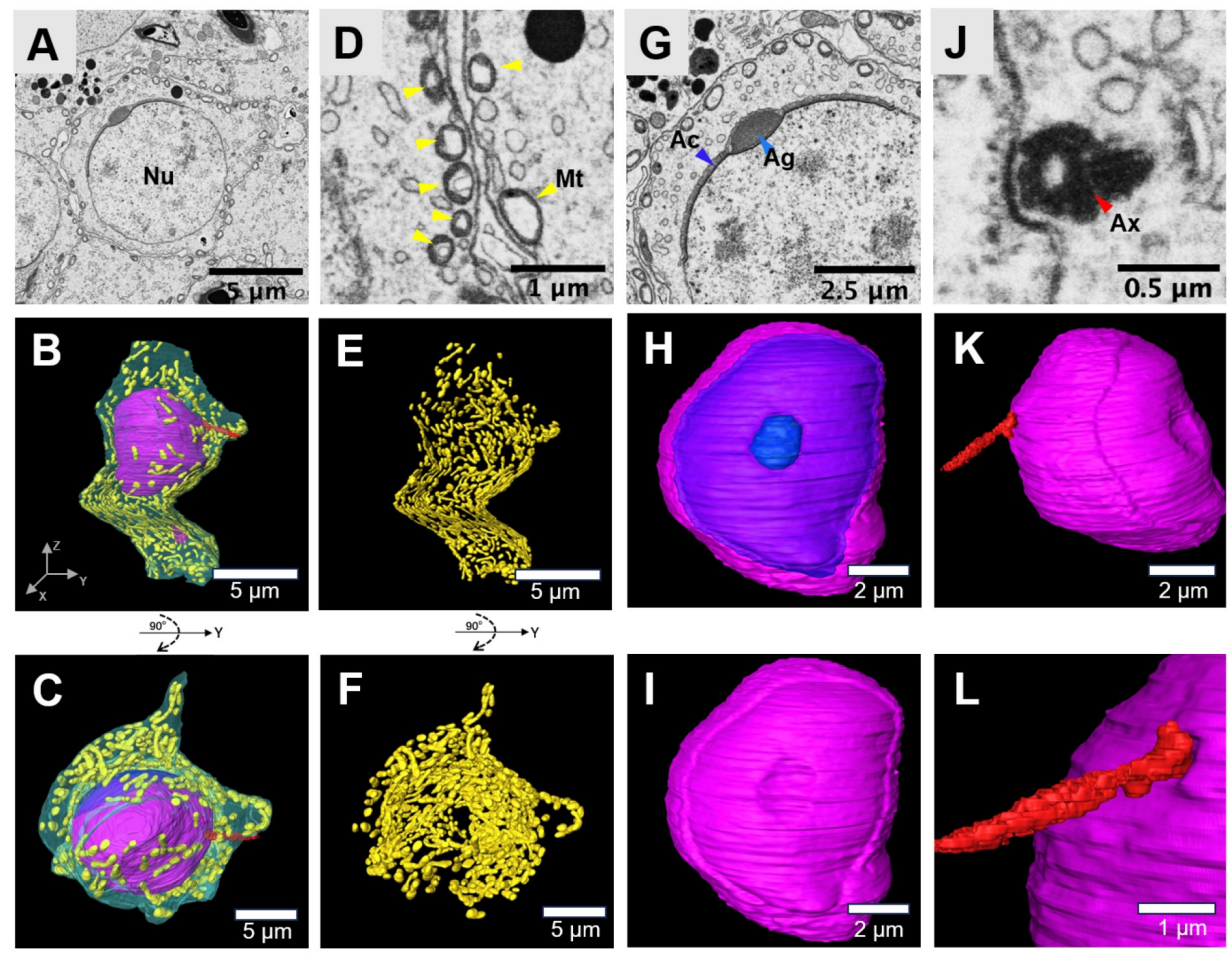
Figure 3 Three-dimensional morphology of the spermatid at the acrosome cap stage (A) Typical SEM image of a spermatid at the acrosome cap stage. Scale bar: 5 μm. (B,C) 3D reconstruction of the cell membrane (transparent merald green), mitochondria (yellow), acrosome cap (light blue), acrosomal granule (dark blue), axoneme (red) and nucleus (purple) of the spermatid. Scale bar: 5 μm. (D) SEM image of the mitochondria in the spermatid. Scale bar: 1 μm. (E,F) 3D reconstruction of the mitochondria. Scale bar: 5 μm. (G) SEM image of the acrosome cap. Scale bar: 2.5 μm. (H,I) 3D reconstruction of the acrosome cap, acrosomal granules and nucleus. Scale bar, 2 μm. (J) SEM image of the axoneme. Scale bar, 0.5 μm. (K,L) 3D reconstruction of the axoneme. Scale bar: 2 μm (K), 1 μm (L). Mitochondria, Mt; acrosome cap, Ac; acrosomal granule, Ag; axoneme, Ax; nucleus, Nu.

In contrast to spermatids in the Golgi phase, spermatids at the acrosome cap stage undergo elongation ( Figure 3A–C). Concurrently, many mitochondria, which appear swollen and condensed, align along the plasma membrane ( Figure 3D–F). The acrosomal granule enlarges and starts to spread across the nucleus envelope, eventually forming a cap that covers approximately one-third of the nucleus surface ( Figure 3G–I). This cap transforms into a very thin layer. Some of these mitochondria relocate toward the axoneme, contributing to the formation of the mitochondrial sheath ( Figure 3J–L). Lipid droplets are sporadically distributed throughout the cytoplasm ( Figure 3A).

### Dimentional morphology of spermatids in the acrosomal phase

**Figure 4:**
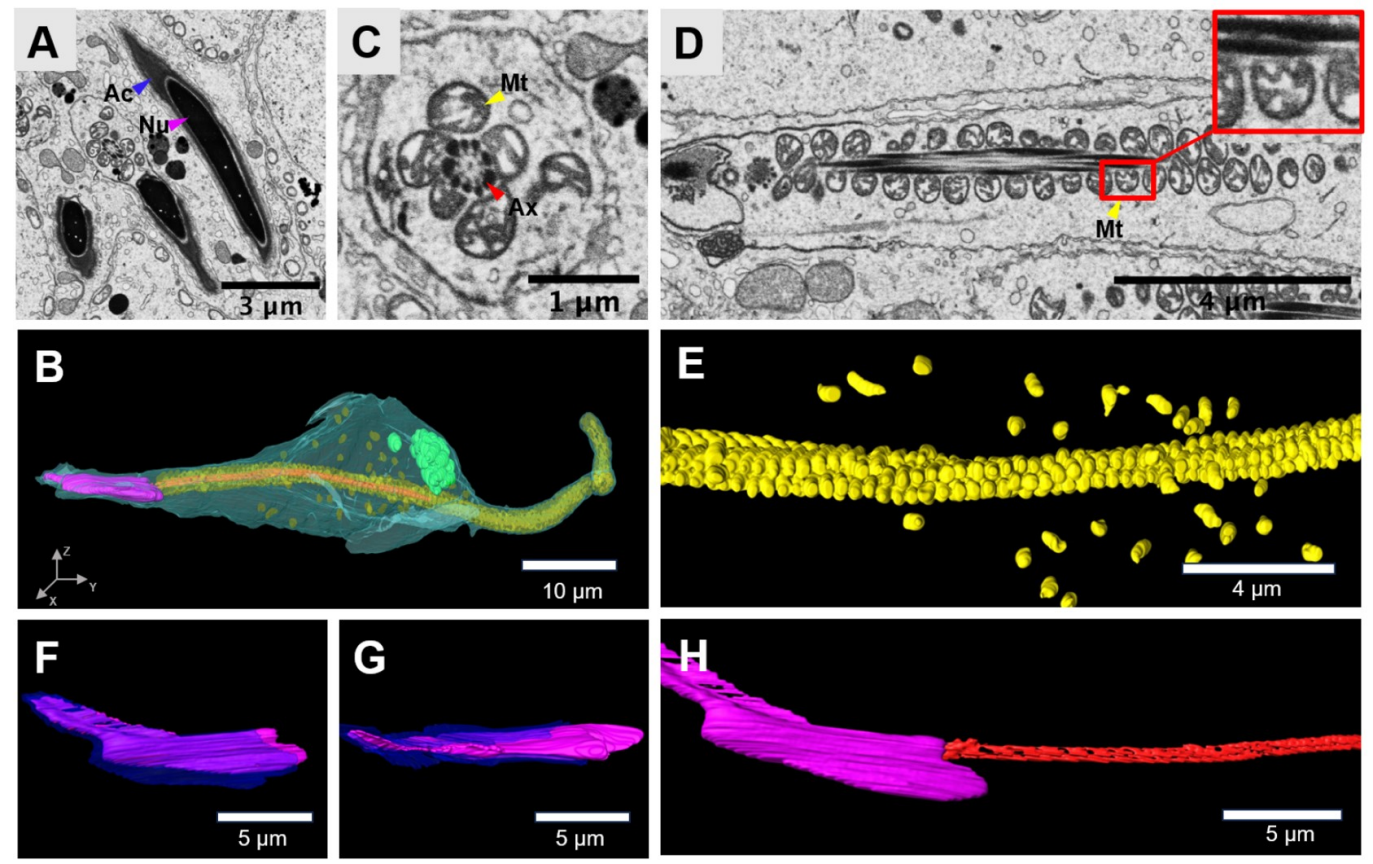
Figure 4 Three-dimensional morphology of the spermatid in the acrosomal phase (A) Typical SEM image of a spermatid in the acrosomal phase. Scale bar: 3 μm. (B) Three-dimensional reconstruction of the cell membrane (transparent yellow), mitochondria (yellow), acrosome cap (blue), lipid droplets (green), axoneme (red) and nucleus (purple) of the spermatid. Scale bar: 10 μm. (C,D) SEM images of the mitochondrial sheath. Scale bar: 1 μm (C), 4 μm (D). (E) 3D morphology of the mitochondrial sheath. Scale bar: 4 μm. (F,G) 3D reconstruction of the acrosome cap and nucleus. Scale bar: 5 μm. (H) 3D reconstruction of the axoneme. Scale bar: 5 μm. Mitochondria, Mt; acrosome cap, Ac; acrosomal granule, Ag; axoneme, Ax; nucleus, Nu.

The acrosomal phase is characterized by significant changes in spermatid morphology and the migration of the acrosome ( Figure 4A,B). As spermatids elongate, their cytoplasm thins, their nuclei condense and elongate, and the acrosome reorients itself. Excess cytoplasm and organelles, such as vesicles and ribosomes, are eliminated from the spermatid. The mitochondria are primarily concentrated in the middle section of the sperm tail, where they coil around the central axoneme in a spiral pattern, eventually forming a mitochondrial sheath structure ( Figure 4C–E). Some mitochondria are also sporadically distributed throughout the cytoplasm. Some mitochondria near the central axoneme attach to it in a comb-like shape, potentially providing kinetic energy to the central axoneme ( Figure 4D). This process is crucial for the successful development and function of sperm, including motility, which is a major determinant of male fertility. The acrosome forms an apex at the anterior part of the spermatid, whereas the remainder migrates to the ventral surface of the spermatid nucleus ( Figure 4F–H).

### Dimentional morphology of the chromatoid body

**Figure 5:**
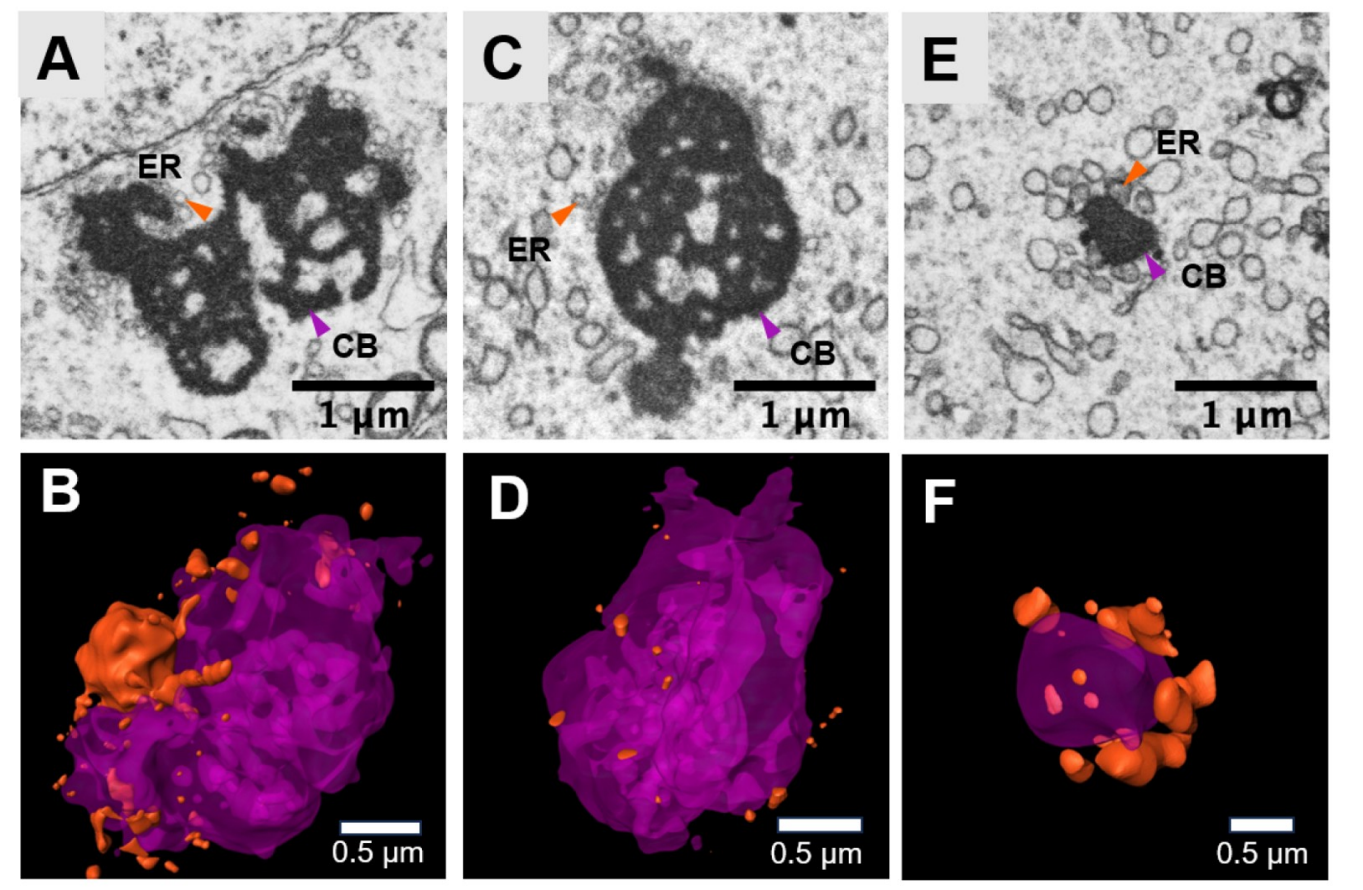
Figure 5 Three-dimensional reconstruction of the chromatoid body in the spermatid at different stages (A,B) Typical SEM image and 3D reconstruction of the chromatoid body at the Golgi-phase stage. (C,D) Typical SEM image and 3D reconstruction of the chromatoid body at the acrosome cap stage. (E,F) Typical SEM image and 3D reconstruction of the chromatoid body at the acrosomal phase stage. A, C and E, scale bar: 1 μm; B, D and F, scale bar: 0.5 μm.

The chromatoid body (CB) is a stage-specific cytosolic structure of the spermatid. Electron microscopy revealed that the CB has a high electron density and appears as a black cloud-like structure with fine vesicles at the periphery of the structure ( Figure 5). The CB stores mRNAs in a translationally repressed state, providing the spermatids at a specific developmental stage with the required proteins, among others. At different developmental stage, the morphology of the CB is different. At the Golgi-phase stage, the CB is unconsolidated and surrounded by ER ( Figure 5A,B). During the acrosome cap stage, the CB is condensed ( Figure 5C,D). At the acrosomal phase stage, the CB is more condensed and smaller compared with the structure in Golgi-phase stage and acrosome cap stage ( Figure 5E,F).

## Discussion

For the first time, we successfully developed a comprehensive pipeline to reconstruct mitochondria and other organelles in the testis via the ATUM-SEM method. ATUM-SEM imaging serves as a potent instrument for the examination of the ultrastructures of three-dimensional cells. This technique can precisely depict the ultrastructural transformations that cells and organelles undergo during the process of spermatogenesis. This innovative approach has resulted in an unprecedented 3D model that provides a detailed map of the testis sample. Moreover, this 3D model facilitates a systematic analysis of the distribution and abundance of various organelles. It also enables the quantification of the membrane contact sites (MCSs) between different organelles.

Our ATUM-SEM results demonstrated that during the round spermatid stage, acrosome structures begin to form, covering the anterior end of the sperm nucleus. Acrosomes are believed to originate from the fusion of pre-acrosomal vesicles that emerge from the trans face of the Golgi apparatus, as described previously [ 19, 20]. In spermatids at the Golgi stage, we observed the initial convergence of vesicular structures to form an acrosomal vesicle. Some of these structures may represent organelles such as the endoplasmic reticulum, which will require further refinement for accurate classification. However, vesicular structures clearly fused with the acrosomal vesicle, confirming that vesicle fusion is indeed a mechanism of acrosomal vesicle formation. During the round spermatid stage, mitochondria are predominantly distributed along the periphery of the cell membrane. As spermatogenesis progresses, mitochondria migrate toward the sperm tail, contributing to the formation of the mitochondrial sheath and the sperm tail itself. Moreover, some mitochondria near the central axoneme did not exhibit the typical round or ovoid morphology but instead exhibited a “comb” shape. Similarly, we reconstructed the chromatoid body in the cytoplasm of sperm cells, which is a special structure that appears during the development of male germ cells. There are reports that CB are derived from the nucleus [21] or nucleolus [22], but some studies have shown that CB are derived from the dense material produced by mitochondria during the late pachytene stage of the first meiotic division [ 23, 24]. The CB can store messenger ribonucleic acid (mRNA) in a transcriptionally repressed state. These mRNAs can be activated for translation at specific developmental stages.

In summary, we generated three-dimensional models of spermatids at three distinct stages. Our research offers insights into the three-dimensional reconstruction of spermatids, including a portion of the organelles found in testicular samples. We also delve into the ultrastructures of these organelles. Our findings present novel technical guidance for biochemical and functional studies of germ cells.

## Supporting information

highlight
