# Supplementary material for: Three-dimensional reconstruction of rat sperm using volume electron microscopy: Morphological process of spermiogenesis revealed by ATUM-SEM
Source: Acta Biochim Biophys Sin (Shanghai). 2024 Sep 3;56(11):1699–705. doi: 10.3724/abbs.2024144 (PMC11659773; doi:10.3724/abbs.2024144)
Supplement: highlight [file highlight.docx]

For the first time, we give a whole picture of the morphological process of spermiogenesis via ultrathin sections-scanning electron microscopy.

1). The 3-D structure obtained by ATUM-SEM provides intricate ultrastructural details during spermiogenesis, including the formation of the acrosome and the generation of mitochondrial sheets, among other processes.

2). The acrosomal vesicles, derived from the Golgi apparatus, converge and elongate along the spermatid nucleus. These vesicles then attach to the nucleus via a cap-like structure, thereby defining the head side of the spermatozoa.

3). During acrosomal phase, the mitochondria primarily concentrate in the middle section of the sperm tail and coil around the central axoneme in a spiral pattern, eventually forming a mitochondrial sheath structure.
